# Long Term Remission and Cardiac Toxicity of a Combination of Ipilimumab and Nivolumab in a Patient With Metastatic Head and Neck Carcinoma After Progression Following Nivolumab Monotherapy

**DOI:** 10.3389/fonc.2019.00403

**Published:** 2019-05-15

**Authors:** Katjana S. Schwab, Glen Kristiansen, Alexander Isaak, Stefanie E. A. Held, Annekristin Heine, Peter Brossart

**Affiliations:** ^1^Department of Internal Medicine 3, University Hospital Bonn, Bonn, Germany; ^2^Institute of Pathology, University Hospital Bonn, Bonn, Germany; ^3^Department of Radiology, University Hospital Bonn, Bonn, Germany

**Keywords:** nivolumab ipilimumab, head neck cancer, immunotherapy, Takotsubo cardiomyopathy (TTC), cardiac toxicity

## Background

The prognosis of recurrent and/or metastatic squamous cell carcinoma of the head and neck (HNSCC) is very poor and treatment options are limited. Patients suffering from HNSCC tumor progression within 6 months after platinum-based chemotherapy have a median survival of 6 months or less (1, 2).

Nivolumab, a programmed death 1 (PD-1) inhibitor, is approved for the treatment of HNSCC being refractory or progressive under platinum based therapy (3). Recent studies showed a significant response of the combination of nivolumab and the cytotoxic lymphocyte antigen 4 (CTLA4) antibody ipilimumab in different tumor types (4, 5). Ongoing trials elaborate the therapeutic success of this combination in HNSCC (NCT03406247, NCT02741570, NCT02919683, NCT03620123, NCT02823574).

Thus, there is limited data concerning the efficacy of this innovative therapeutic regimen in HNSCC, especially after progression following monotherapy with a PD-1 inhibitor. A single case has reported a remarkable response to the combination of nivolumab and ipilimumab in a patient with platinum refractory HNSCC without previous immunotherapy (6).

### Case Presentation

A 69-year old male patient with initial diagnosis of a squamous cell cancer of the lower lip in 2013 underwent complete resection including plastic reconstruction of the lip and vermillionectomy including submental lymphnode resection on the left and right side. The histology demonstrated an infiltration of a moderately differentiated squamous cell cancer of the lip incorporating muscle invasion. The margins were negative. There were no signs of metastases. So the initial tumor stage due to TNM classification was pT1,pN0 (0/6),L0,V0, M0,R0, G2 (moderately differentiated). Due to the staging, no adjuvant therapy was administered.

In May 2015, the patient presented with a swelling in the right cheek. The subsequent biopsy confirmed a relapse consisting of a submandibular lymph node metastases on the right side. A neck dissection level I-V on the right and level I-III on the left side was performed followed by local radiotherapy (63 Gy on right, and 54 Gy on the left side) until August 2015. Therapy was well-tolerated without any relevant clinical symptoms. On routine follow-up in December 2015, a local relapse with new lymph node manifestations on the right side intra- and retro parotideal was confirmed. A whole body CT-scan showed no signs of metastases. A complete resection of the tumor manifestation was considered infeasible and, so, a systemic chemotherapy with cisplatin, 5-FU and cetuximab was initiated. After four cycles, a MRI-scan of the neck revealed progressive disease.

**Figure 1:**
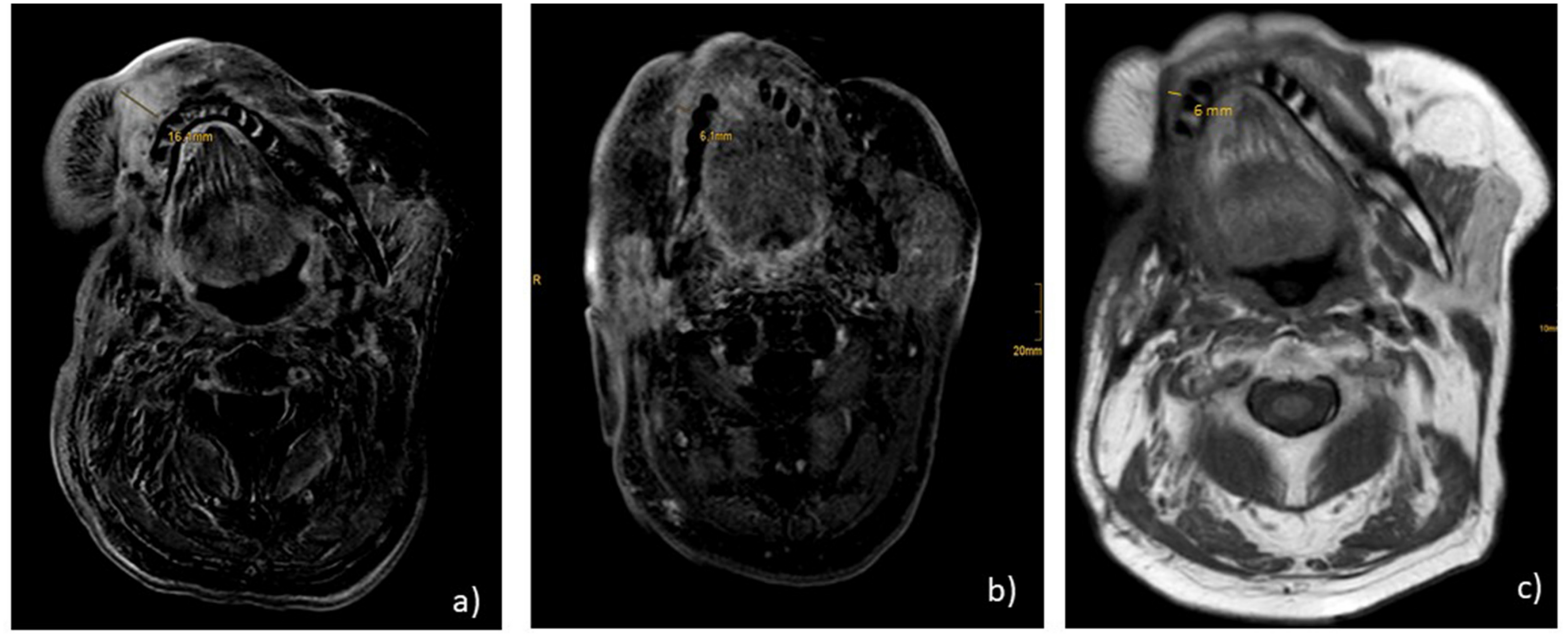
MR.I T1 **(a)** progression under nivolumab monotherapy before start of nivolumab and ipilimumab; **(b)** partial remission under nivolumab and ipilimumab after 2 cycles; **(c)** ongoing stable disease 2 years after start of combination therapy and 7 months after end of treatment.

Immunohistochemical staining of the lymphatic node metastases from May 2015 revealed that 25% of the tumor cells were positive for PD ligand 1 (PD-L1), whereas PD-1 expression was negative. Based on these findings, therapy with nivolumab was started in April 2016 (3 mg/kg every 2nd week). After 6 administrations, restaging with CT and MRI revealed no change of the tumor extension (stable disease), and, therefore, nivolumab was continued. Therapy was well-tolerated and no side effects occurred. Unfortunately, in August 2016 after 11 administrations of nivolumab local progression was confirmed by routine follow up and radiologic scans (Figure 1a). There were no signs of metastases. In addition, no surgical approach was reasonable. Due to the lack of other therapeutic options, a combination with nivolumab and ipilimumab was initiated (nivolumab 3 mg/kg on days (d) 1,14,28, and ipilimumab 1 mg/kg on d1 following every 42 days). In January 2017, following two cycles, MRI of the neck confirmed a partial remission (size reduction of more than 50%) (Figure 1b) and therapy was continued. This treatment was well-tolerated. Apart from a fatigue syndrome, no symptoms due to immunotherapy was reported by the patient. In the following clinical schedules, MRI examinations after 4 and 6 cycles revealed stable disease, without any change of tumor size. At this point, therapy was well-tolerated, the patient was able to carry on normal activity with minor symptoms of disease, such as fatigue syndrome (Karnofsky-Index of 90%).

After the 7th cycle (September 2017), the patient presented with progressive shortness of breath, weakness, and confusions. Laboratory findings revealed an acute renal failure grade III with hypercalcaemia probably associated with immunotherapy. Thus, therapy with nivolumab and ipilimumab was interrupted and oral steroid therapy was initiated with prednisolone 100 mg/d (1 mg/kg/d). After a few days, the clinical condition improved, the confusion disappeared, and the renal function normalized. Prednisolone was reduced stepwise to 75 mg after 7 days.

Three days after discharge, the patient was rehospitalized triggered by acute chest pain and shortness of breath. NSTEMI—constellation was diagnosed and a coronary angiography was performed, showing no signs of relevant coronary artery stenosis. A transthoracic echocardiography revealed a reduced cardiac ejection fraction of 36% (ejection fraction before start of immunotherapy was >60%) accompanied by apical balloning. A cardiac MRI detected the presence of a Tako-Tsubo cardiomyopathy, possibly caused by the immunotherapy with ipilimumab and nivolumab. Of note, the patient reported no history of cardiac co-morbidities.

Consequently, heart failure therapy and treatment with prednisolone 50 mg/d was initiated. Immunotherapy was discontinued until December 2017. In January 2018, echocardiography showed a normalization of cardiac function with an ejection fraction of >60%. At this time, steroid dosage was reduced to 5 mg per day. Accordingly, monotherapy with nivolumab was re-introduced and steroid therapy was stopped completely. In March 2018, after MRI showed on-going stable disease, immunotherapy was stopped according to the patient’s demand. Up to now, the patient remains in very good physical condition (Karnovsky of 90%). Follow up CT and MRI scans in July and October 2018 confirmed ongoing stable disease without any signs of tumor progression (Figure 1c). Consequently, therapy has not been reinitiated, yet.

### Investigations

**Figure 2:**
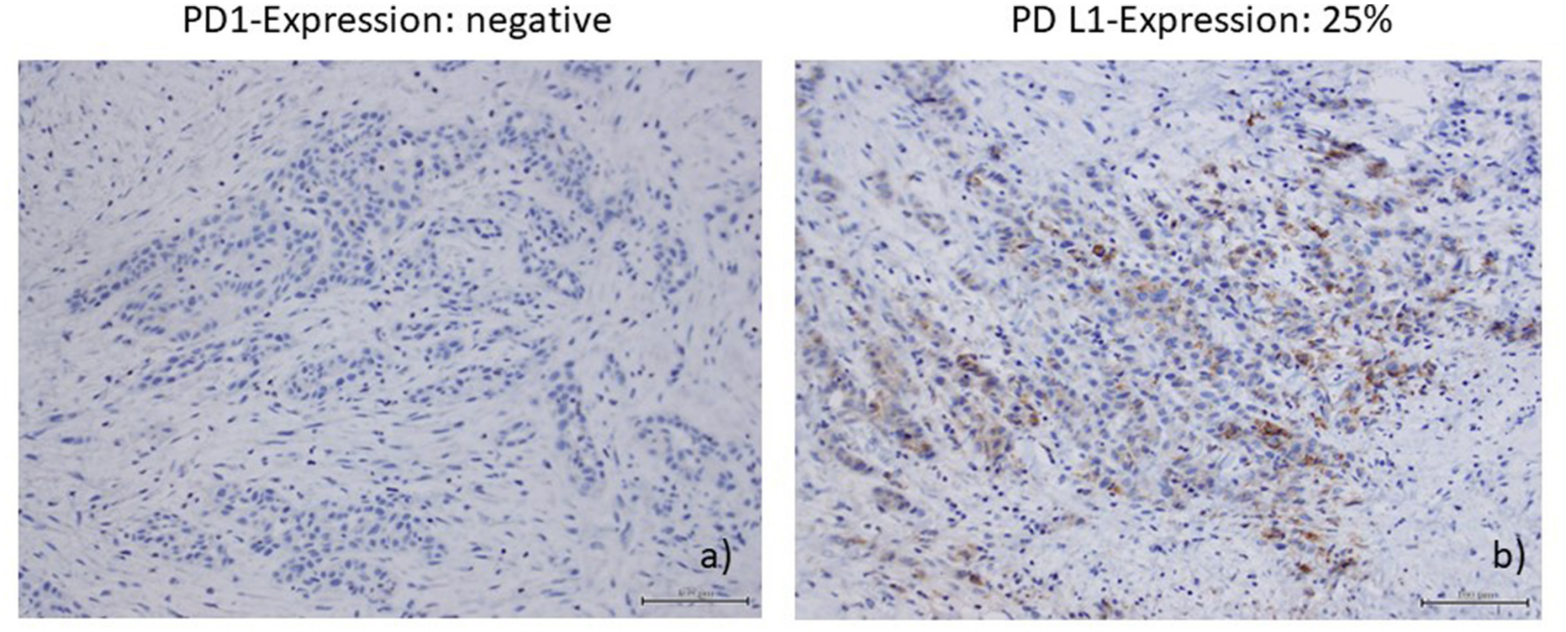
**(a)** PDl-expression (Autostainer Benchmark Ultra; Ventana, antibodies: PDl-Clone NAT105, Abeam, UK); **(b)** PD-Ll-expression (PD-Ll-Clone 22C3, Dako, Denmark).

Immunohistochemical staining of the lymphatic node metastases from May 2015 showed that 25% of the tumor cells were positive for PD ligand 1 (PD-L1), whereas PD-1 expression was negative (Figures 2a,b). PD1 and PD-L1 were immunohistochemically analyzed (Autostainer Benchmark Ultra; Ventana, antibodies: PD1-Clone NAT105, Abcam, UK, and PD-L1-Clone 22C3, Dako, Denmark). Furthermore, there was an increased infiltration of CD8–positive T-lymphocytes of the tumor microenvironment, 150 cells/HPF.

In the peripheral blood leukocytes, neutrophil count and lymphocytes were evaluated before, during and after immunotherapy. Before start of nivolumab monotherapy leukocytes were 8.8 G/l, neutrophils were 6.9 G/l, and lymphocytes were 0.57 G/l. When combination therapy with ipilimumab was initiated, leukocytes were 9.4 G/l, neutrophils were 7.0 G/l, and lymphocytes were 0.8 G/l. After start and during prednisolone therapy there was an increase of leukocytes (10.5 G/l) and neutrophils (8.93 G/l) whereas lymphocytes decreased (0.5 G/l). After termination of prednisolone administration, there was a decrease of leukocytes (7.3 G/l) and neutrophils (5.2 g/l) and lymphocytes increased again (0.85 G/l).

## Discussion

Nivolumab monotherapy in the treatment of recurrent and metastatic HNSCC has increased the overall survival time as well as disease-free survival (3). In contrast, promising therapeutic options after progression following nivolumab monotherapy are lacking.

The current report provides evidence of an unexpected and durable response to combination of nivolumab and ipilimumab after treatment failure of established, nivolumab monotherapy. To our knowledge, this is the first case report of a long term remission after therapy with nivolumab and ipilimumab in HNSCC for more than 2 years in a patient who relapsed upon treatment with nivolumab monotherapy.

Despite important clinical benefits, immunotherapy is known to cause serious side effects and toxicities (7). In particular, studies reported the occurrence of immunotherapy related myocarditis after nivolumab monotherapy (8–11). Such a life threatening condition, presented as a Tako-Tsubo state, was also seen in our patient. Consequently, treating oncologists have to be aware of any possible serious side effect of immunotherapy to provide acute diagnostics and therapy.

Taking into account the serious toxicity associated with immunotherapy, further investigations are necessary to evaluate possible predictors of response to immunotherapy.

Recently, Ho et al. published an association between pre-treatment lymphocyte count and response to PD-1 inhibitors in head and neck cancer (12). Patients with an absolute lymphocyte count (ALC) of <0.6 G/l showed a shorter progression free survival (PFS). This is in accordance with the trend of the absolute lymphocyte count in our patient, who showed an ALC of 0.8 G/l before start of combination therapy with ipilimumab and nivolumab, leading to a partial response after two cycles of therapy. However, ALC as potential predictor of response to immunotherapy remains the subject of further investigations.

In conclusion, combination of ipilimumab and nivolumab reveals an option in refractory and/or metastastic squamous cell cancer of the head and neck, even in patients with progressive disease under nivolumab monotherapy. Nevertheless, the potentially life threatening side effects of this therapy has to be taken into account and patients must be surveilled carefully during this novel therapeutic approach.

## Ethics Statement

Written informed consent was obtained from the patient for the publication of this case report and any potentially-identifying information and images.

## Author Contributions

All authors listed have made a substantial, direct and intellectual contribution to the work, and approved it for publication.

### Conflict of Interest Statement

The authors declare that the research was conducted in the absence of any commercial or financial relationships that could be construed as a potential conflict of interest.
